# Advances in molecular and cell therapy for immunotherapy of cholangiocarcinoma

**DOI:** 10.3389/fonc.2023.1140103

**Published:** 2023-03-29

**Authors:** Li-ming Zhao, An-da Shi, Yan Yang, Zeng-li Liu, Xiao-Qiang Hu, Li-Zhuang Shu, Yong-chang Tang, Zong-li Zhang

**Affiliations:** ^1^ Department of General Surgery, Qilu Hospital, Shandong University, Jinan, China; ^2^ Department of General Surgery, Shanxian Central Hospital, Heze, China; ^3^ Department of General Surgery, Qilu Hospital (Qingdao), Shandong University, Jinan, China

**Keywords:** immunotherapy, PD-1/PD-L1 inhibitor, CTLA4 inhibitor, molecular therapy, cell transplantation, vaccines, cell therapy, cholangiocarcinoma

## Abstract

Cholangiocarcinoma (CCA) is a highly malignant tumor of the hepatobiliary system that has failed to respond to many traditional therapies to a certain extent, including surgery, chemotherapy and radiotherapy. In recent years, the new therapeutic schemes based on immunology have fundamentally changed the systemic treatment of various malignant tumors to a certain extent. In view of the immunogenicity of CCA, during the occurrence and development of CCA, some immunosuppressive substances are released from cells and immunosuppressive microenvironment is formed to promote the escape immune response of its own cells, thus enhancing the malignancy of the tumor and reducing the sensitivity of the tumor to drugs. Some immunotherapy regimens for cholangiocarcinoma have produced good clinical effects. Immunotherapy has more precise characteristics and less adverse reactions compared with traditional treatment approaches. However, due to the unique immune characteristics of CCA, some patients with CCA may not benefit in the long term or not benefit at all after current immunotherapy. At present, the immunotherapy of CCA that have been clinically studied mainly include molecular therapy and cell therapy. In this article, we generalized and summarized the current status of immunotherapy strategies including molecular therapy and cell therapy in CCA in clinical studies, and we outlined our understanding of how to enhance the clinical application of these immunotherapy strategies.

## 1 Introduction

Cholangiocarcinoma (CCA) is a highly malignant tumor of the digestive system with a poor prognosis that poses a significant therapeutic challenge (1). Based on their anatomical distribution, they are classified as intrahepatic (iCCA), perihilar (pCCA), or distal (dCCA) (2). Treatment outcomes are extremely poor due to early local invasion, susceptibility to multiple metastases, poor effectiveness of most treatment options, and frequent drug resistance (3, 4). Moreover, due to the lack of early specific molecular markers to detect cholangiocarcinoma, most cholangiocarcinoma is already at an advanced stage when detected (5, 6). Despite the efforts of many studies, clinical transformation remains stagnant, making the treatment of cholangiocarcinoma more difficult. Surgical resection is the only potentially curative treatment option for CCA. However, only approximately 15% of patients with iCCA have a removable stage at initial diagnosis, and the postoperative recurrence rate is more than 60% (7, 8). Systemic chemotherapy is one of the recognized first-line treatments for patients with metastatic and advanced cholangiocarcinoma, but the prognosis after this treatment remains poor, with 5-year survival rates below 40% (9, 10). Radiotherapy as adjuvant or palliative treatment has a limited impact on the survival of patients with cholangiocarcinoma (11–13). Currently, only a minority of CCA patients have the opportunity to undergo radical resection. Nonetheless, median survival beyond 30 months is challenging even with radical resection (14). Especially for patients with advanced CCA, there are few effective treatments that can improve clinical outcomes. Therefore, there is an urgent need to develop more effective treatment options to improve the clinical prognosis of CCA patients, especially those with advanced CCA.

Over the past decade, tumor immunotherapy has emerged as an emerging treatment for various cancers, including melanoma, urothelial tumors, non-small cell lung cancer, and liver cancer (15). Tumor immunotherapy refers to using immunological principles and methods to activate immune cells *in vivo* and enhance the body’s anti-tumor immunity, so as to specifically remove minimal residual tumor lesions, slow down tumor growth, and alleviate immune tolerance (16). In other words, the goal of cancer immunotherapy is to break the mechanism of cancer immune escape, thereby reawakening immune cells and destroying tumor cells. Due to its minimal side effects and excellent therapeutic effect, it is gradually becoming the future development direction of tumor treatment, and is known as the fourth most important cancer treatment method after surgery, radiotherapy and chemotherapy (17–19). In a word, from the perspective that CCA is an immunogenic cancer, immunotherapy will provide a promising treatment strategy to improve the survival benefit of patients with CCA.

## 2 Background on immunotherapy for cholangiocarcinoma

Cholangiocarcinoma is an immunogenic tumor (16). It is characterized by fewer tumor parenchyma cells and more tumor interstitial cells compared with other solid tumors (20). It has a microenvironment controlled by inflammation and rich immune cells, mainly composed of T and B lymphoid cells, macrophages, neutrophils, natural killer cells, and other subsets of immune cells (21). On the one hand, in the innate immune system of cholangiocarcinoma, macrophages, as the first line of defense, have strong heterogeneity and play a crucial role in CCA (22, 23). Macrophage infiltration is closely related to angiogenesis and increased regulatory T cell (Treg) infiltration (24). Dendritic cells (DCs) belong to antigen presenting cells (APCs), which are the sentinels of the human immune system. They can keenly capture the small difference between tumor cells and normal cells, and transmit this difference to the armed police T-lymphocytes in the human immune system, which is crucial in activating the adaptive immune response (25). On the other hand, in adaptive immune response of cholangiocarcinoma, tumor infiltrating lymphocytes (TIL) are a highly heterogeneous population, including CD8 + T cells, CD4 +T cells, B cells and Treg (26, 27). They play an important role in CCA immune surveillance and elimination of tumor cells. CD8 + T cells is one of the main effector cells of tumor immune adoptive therapy. They bind to target cells through the receptor TCR on their membranes, releasing various lysosomes, thus promoting the dissolution and death of target cells (28).

The immune microenvironment in CCA could be characterized by excessive secretion of cytokines and chronic persistent inflammatory infiltration, which could induce the proliferation of tumor bile duct cells (29, 30). The high-density infiltration of tumor associated macrophages may play a role in supporting the metastasis and degradation of extracellular matrix (31). This helps CCA cells escape from immune system (32). Therefore, this immunological characteristic of cholangiocarcinoma creates an unfavorable element for the host’s adaptive immune response to CCA. Our better understanding of the immune microenvironment of cholangiocarcinoma is helpful to improve the prognosis of CCA patients through immunotherapy.

## 3 Molecular therapy

The main principle of molecular therapy is to inject specific molecular agents into the patient’s body to regulate the body’s specific immune response. The main agent used is immune checkpoint inhibitors (ICIs). The related content of ICIs is an attractive issue at the territory of cancer treatment, which has made vast progress in the treatment of cancers originated from different organizations and organs (33). ICIs mainly include (34): (1) Programmed cell death protein 1 (PD-1) inhibitors. It is represented by pembrolizumab and nivolumab. (2) PD-L1 inhibitors. It is represented by durvalumab, avelumab and atezolizumab. (3) Cytotoxic T lymphocyte-associated antigen 4 (CTLA-4) inhibitors. It is represented by tremelimumab and ipilimumab. CCA cells can use these inhibitors to limit or evade antitumor immune responses, including by affecting the synthesis of immune checkpoint associated proteins (35). At present, numerous clinical studies of ICIs related to iCCA, pCCA and dCCA are performing. Several results of clinical trials and cases have shown that patients with certain genotypes of CCA may benefit from treatment with ICIs.

### 3.1 PD-1 and PD-L1 inhibitors

PD-1 is a membrane-spanning protein located on the surface of T lymphocytes that eliminates antitumor immune reactions and facilitates cancer immune evade from cytotoxic T cells in the course of carcinogenesis, while PD-L1 is a PD-1 ligand molecule expressed by tumor cells. PD-1 is related to immune responses and the immune microenvironment of cholangiocarcinoma (36). In Tumor Microenvironment (TME), PD-1 and PD-L1 molecules combine to induce T lymphocyte failure, leading to tumor immunosuppression. Hence, blocking the PD-1/PD-L1 signal axis by inhibiting the combination of them can gradually improve the adaptive immune system, reverse the phenomenon of immune suppression, and restore the response of cancer cells to T cells. Using monoclonal antibodies to cut off the PD-1/PD-L1 pathway has shown a good effect with long-lasting responses and prolonged survival in patients with some cancer types including CCA. What’s more, these reactions usually last for many years or never disappear and do not cause severe toxicity for vast majority of patients (15). Based on this, we believe that it is a potential and promising therapeutic strategy for CCA.

In fact, the response of patients with different molecular types of tumors to immunotherapy is inconsistent. At present, it is believed that PD-L1, MMR, MSI, and high TMB positive of some tumor patients may have a good response to immunotherapy. This is because a high level of mutation‐associated neoantigens can be distinguished by immune cells (37). At the same time, the study found that, with the continuous increase of TMB, the progression free survival (PFS) and objective response rate of CCA patients treated with ICIs may increase (38). There are some case reports have showed that patients with irresectable or recurrent metastatic biliary tumor have achieved partial remission (PR) or complete remission (CR) through ICIs treatment. Therefore, these successful individualized treatment schemes provided important guidelines for the management of patients with last-stage cholangiocarcinoma. High expression of immune checkpoints in CCA patients is related to poor prognosis, especially with short relapse free survival and overall survival. Due to the immune escape mechanism, the high expression of PD-L1 may be related to the rapid progress and poor prognosis of the tumor. Meta-analysis of eleven researches about cholangiocarcinoma and PD-1 found that the expression of PD-L1 in cholangiocarcinoma cancer cells went together with TNM staging. Overexpression of PD-L1 predicted poor overall survival rate (OS), and overexpression of PD-L1 also predicted short disease-free survival (DFS) period (39). In addition, similar conclusions have been found in other studies that high lymphatic metastasis, TNM stage and poor prognosis are closely related to PD-L1 positive (40, 41). Another study demonstrated by section staining that a high count of CD8+T cells in tumor parenchyma and stroma resulted in worse OS and DFS, and a high expression of PD-L1 resulted in worse overall survival (OS). Immunosuppression of PD-L1 may be a potential treatment for CCA patients who are unable to undergo surgery (42).

One study showed that PD-L1 is mainly expressed by inflammatory cells in tumors with dense lymphocyte infiltration. Their results suggested that CCA patients who have dense intratumoral lymphocytic infiltration may be a suitable treatment character for PD-L1/PD-1 inhibitors (43). Else, a study showed tumors could create their own tumor microenvironment to enhance PD-L1 expression, and then relying on this pathway to achieve immune escape in CCA with dense lymphocytic infiltrates (44). Driven by these studies, single-inhibitor drugs have gradually entered clinical use, and two of the most representative drugs in CCA are: pembrolizumab and Nivolumab (Opdivo). In 2017, the FDA recommended the utilization of pembrolizumab to treat cancers including cholangiocarcinoma (45). National Comprehensive Cancer Network (NCCN) guidelines recommended the use of pembrolizumab as one of the treatment options for tumors (including biliary malignancies) with defective microsatellite instability (MSI) or mismatch repair (MMR) characteristics. A study of defective mismatch repair cancers treated with pembrolizumab in cholangiocarcinoma patients has showed an excellent objective response rate (ORR=40.9%) (46). Nivolumab is an immune checkpoint inhibitor that essentially exploits its PD-1 -binding properties to IgG4 immunoglobulin. In a small sample size study of cholangiocarcinoma tumor samples, it was found that nivolumab expression could be detected at different levels in tumor microenvironment, both in tumor cells and immune cells (35). And it has been demonstrated that PD-1 expression increased in CCA patients with CD4+ and CD8+ tumor‐infiltrating lymphocytes (TILs), and the level of effector cytokines in TILs was positively correlated with nivolumab (47). One phase II trial showed that TILs could play a significant role in adjusting the interaction between ICI and tumor microenvironment (45). The research also showed that this inhibitor with radiotherapy or chemotherapy in CCA treatment have been examined and shown a good therapeutic prospect (45). These data showed that nivolumab might provide a novel therapeutic choice for cholangiocarcinoma patients.

Certainly, with more and more studies, we have found that ICIs combination therapy is better than ICIs monotherapy. Clinical trials of anti-PD-1/PD-L1 medicine combined with other therapies for CCA patients are currently in full progress at the moment. The study found that nivolumab is superior to single drug immunotherapy when used in combination with CTLA-4 inhibitor ipilimumab (41). In addition, in the model of CCA subcutaneous tumor, the combined use of verteporfin and anti-PD-1antibodies down regulated the tumor burden compared with the use of two medicines alone (48). Another study showed that anti-PD-1 immunotherapy combined with radiotherapy is an up-and-coming one. In this study, Liu et al. presented a 68-years-old iCCA patient in advanced stage who is not suitable for chemotherapy, after six cycles of ICIs therapy, the primary tumor narrowed but new lung and lymph gland metastasis were found, which indicated a mixed response. Radiotherapy was then started, concomitant with continuing ICIs immunotherapy. The combination treatment ultimately led to complete remission of primary tumors and all metastatic tumors, and almost no treatment related adverse events occurred (49). Besides, Zhang et al. reported that the first patient with last-stage intrahepatic cholangiocarcinoma with PD-L1 positive and high tumor mutation load was successfully eradicated after using tyrosine kinase inhibitors (TKIs) combined with ICIs immunotherapy to shrink the tumor. In this case, a thirty-eight years old young woman was diagnosed with stage IV iCCA. The test report of this patient indicated that the tumor mutation load was high and the expression of PD-L1 was positive. After receiving seven cycles of PD-1 inhibitor combined with TKIs therapies, the patient underwent radical operation, and it is noteworthy that her postoperative pathological type is well (50). A case report showed a patient with metastatic iCCA who was not sensitive to first-line chemotherapy therefore was included in the phase I study of sintilimab therapy. In addition, the tumor mutation load in this patient was low and with microsatellite instability. The patient was completely relieved after three courses of treatment. Therefore, the author believed that PD-1 inhibitor combination therapy may be a feasible treatment for patients with advanced cholangiocarcinoma who are not sensitive to chemotherapy (51). Zhao reported four cases of intractable advanced CCA patients who managed to control the tumor with anti-PD-1 antibodies together with SBRT (52). A study conducted in patients with unresectable iCCA showed that the efficacy and safety of HAIC (n=58) or TACE (n=39) combined with ICIs immunotherapy or TKIs were more positive than that of HAIC and TACE alone (53). Li et al. found that PD-1 inhibitor combined with S-1 and nab paclitaxel can be used to achieve the transformation treatment of advanced refractory CCA. This combination therapy is safe and effective, and can significantly prolong the survival of patients (54). Previous evidence suggested that PD-1 inhibitors are suitable for patients who have high TMB, high microsatellite instability (MSI-H), deficient mismatch repair (dMMR), and PD-L1 positive expression. But a new study came to a different conclusion. It found that SBRT combined with PD-1 inhibitor can effectively treat patients who have low TMB, MSI, pMMR and PD-L1 expression negative in advanced or recurrent CCA. This finding is of great significance, because it may expand the indications of combined therapy, so that those patients who were not previously suitable for immunotherapy can benefit from immunotherapy (34). Integrating these findings, we believe that PD-1 and PD-L1 inhibitors have a significant role in the treatment of cholangiocarcinoma, and the combination therapy may be one of the important options for patients with advanced CCA.

Of course, the PD-1/PD-L1 treatment of cholangiocarcinoma is extremely complex, and there are also some other studies on this aspect. Because PD-L1 in CCA is predominantly derived from tumor-associated macrophages (TAMs), PD-1 blockade can be enhanced by targeting tumor-associated macrophages and granulocyte-myeloid-derived suppressor cells (55). One study has found that PD-L1 is related to p-ERK, and the down-regulation of KRAS can down-regulate the expression of PD-L1 through this pathway in CCA (56). Based on this, relevant therapeutic strategies can be developed to influence the responsiveness of CCA to ICIs. Else, the study found that stimulation of some antigen-presenting cells (dendritic cells and macrophages) with CD40 agonists can enhance the ICIs effect in CCA (57). In addition, a very important regulator, CMTM4, was identified in CCA patients, which can positively regulate PD-L1 and stabilize PD-L1 by affecting the post-translational biological manifestations (58).

### 3.2 CTLA4 inhibitors

Another important aspect of ICIs treatment is CTLA4 inhibitor. CTLA-4 molecules can be highly expressed on the surface of activated T cells such as Tregs, and interact with its ligands to produce signals that affect the activation and proliferation of T cells (59–62). The CTLA-4 mono-cloning antibody ipilimumab is the first immunosuppressant approved for marketing by the US FDA. One study has found that ipilimumab could cooperate with Nivolumab to significantly improve the clinical outcome of patients with intrahepatic CCA (63). Furthermore, some research teams have conducted two clinical trials to investigate the efficacy of CTLA-4 monoclonal antibody tremelimumab and PD-1 monoclonal antibody durvalumab combined with first-line chemotherapy or TACE to cure advanced unresectable biliary system malignant tumors (41). Of course, these studies are ongoing, and we expect the results will bring good news for the treatment of cholangiocarcinoma.

Researchers have done a lot of work in clinical research related to cholangiocarcinoma immune checkpoint, and we summarized it (**Table 1**). Certainly, it is generally believed that PD1/PD-L1 monoclonal antibody has stronger anti-tumor effect than CTLA-4 monoclonal antibody. Compared with CTLA-4, PD-1/PD-L1 inhibitors produce less toxic and side effects on human body and exhibit better overall efficacy. The phase III head-to-head trial has found that the effectiveness of PD-1 pathway blocking (pembrolizumab, 33%-34%) is better than that of CTLA-4 blocking (ipilimumab, 12%). At the same time, the survival rate (1 year) of PD-1 treatment was higher than that of CTLA-4 (71, 72). Therefore, we believed that PD1/PD-L1 will become an important branch of tumor treatment in the following years.

**Table 1:** Clinical trials related to immune checkpoint therapy in CCA.

Author	Trial phase	Patient	Intervention	ClinicalTrials.gov Identifier
Le et al. (64)	Phase II	MMR-defificient cancers including CCA	Pembrolizumab	NCT01876511
Marabelle et al. (46)	Phase II	High microsatellite instability cancers including CCA	Pembrolizumab	NCT02628067
Kim et al. (65)	Phase II	Biliary tract cancers including CCA	Nivolumab	NCT02829918
Oh et al. (66)	Phase III	Biliary tract cancers including CCA	Durvalumab	NCT03875235
PIHA-PAUL et al. (67)	Phase Ib	Biliary tract cancers	Pembrolizumab	NCT02054806
PIHA-PAUL et al. (67)	Phase II	Biliary tract cancers	Pembrolizumab	NCT02628067
Doki et al. (68)	Phase I	Biliary tract cancers	Durvalumab	NCT01938612
Yarchoan et al. (69)	Phase II	Biliary tract cancers	Atezolizumab	NCT03201458
Xie et al. (70)	Phase II	Biliary tract cancers, predominantly CCA	Tremelimumab + Subtotal Microwave Ablation	NCT01853618
Klein et al. (63)	Phase II	Biliary tract cancers including CCA	Nivolumab + Ipilimumab	NCT02923934
Oh et al. (66)	Phase II	Biliary tract cancers including CCA	Durvalumab ± Tremelimumab + Gemcitabine + Cisplatin	NCT03046862
Kelley et al. (9)	Phase III	Biliary tract cancers including CCA	Pembrolizumab + Gemcitabine + Cisplatin	NCT04003636
G -L et al. (68)	Phase III	Biliary tract cancers including CCA	KN035 + Gemcitabine + Oxaliplatin	NCT03478488

## 4 Cell therapy

Cell therapy of cholangiocarcinoma is mainly to activate the specific or non-specific immune function of the body through cell transplantation or cell vaccine, enhance the body’s immunity, so as to improve the survival of patients with CCA.

### 4.1 Treatment with cell transplantation

Main methods of cell transplantation, such as adoptive immune response cell therapy (ACT) is to isolate cells with good immune activity from cancer patients, perform proliferation and functional verification *in vitro*, and then transplant them into patients, so as to increase the quantity of immune cells against tumor. At present, there are three main methods for this scheme: TIL therapy, chimeric antigen receptor T cell technology (CAR-T) and T cell receptor chimeric T cells (TCR-T) (73). TIL cell therapy refers to the treatment of isolating tumor infiltrating lymphocytes from tumor tissue, which is also a therapy of reinfusion to patients after *in vitro* culture and expansion. The effector cells of TIL therapy are the population with a high proportion of tumor specific T cells and rich diversity, so it has the advantages of multiple targets, strong tumor tendency and invasion, and small side effects (74). TCR-T therapy is a therapeutic method using antigen specific TCR transduced T cells, while CAR-T therapy is a method using CAR gene to apply T cells (75). TCR-T and CAR-T have received extensive attention and research because they can target and distinguish specific cancer cells by expressing unique receptors. And the birth of the two therapies can remove the restriction on whether more high-quality antigen reactive T cells can be obtained from tumor tissues of CCA patients (76).

The success of ACT in treating CCA has been reported in some case studies, but its exact efficacy still needs to be further proved by large sample trials (77, 78). Presently, the reliable evidence for treating CCA patients with ACT is confined to some case reports and occasional series of cases enrolled in a single arm phase II clinical study (79). In the year of 2006, A case report first proposed the clinical effectiveness of a CCA patient who received adoptive cell therapy. In this study, an iCCA patient with lymph node metastasis acquired curative excision and was transplanted with subsidiary CD3-activated T cells added cancer peptide- or lysate-pulsed dendritic cells. After the treatment, the patient has survived for more than 3.5 years (77). In 2014, Rosenberg’s team from the National Institute of Health (NIH) covered an example of a metastatic cholangiocarcinoma. The patient received TIL infusion which was co-cultured with antigen-presenting cells (APCs) transplanted with non-reproductive cell non-synonymous mutations found in the cancer, leading to cancer extinction for more than half a year (78). Some studies on the prognostic significance of TIL treatment for CCA patients show that patients with large number and high density of CD8+T cells show better OS or DFS (80). The study found that the large number of CD8+T cells at the tumor margin was related to OS prolongation. Similarly, higher CD4+T cell density at tumor margin is associated with better OS or DFS, and the distribution of CD8+T cells within and around the tumor also affects the prognosis of CCA patients (74, 78).

Another phase I clinical trial (NCT01869166) showed that CAR-T cell therapy was safe in people with advanced, unresectable biliary cancer. Among 17 evaluable patients, 1 patient achieved complete remission and 10 patients were in stable condition (81). One case reported a CCA patient with mediastinal lymph node metastasis in stage IV, who received Vγ9Vδ2 T cell immunotherapy, the immune function was improved and TNM stage decreased (82). Besides, one phase II clinical trial reported that a CCA patient who participated in the adoptive cell therapy experiment and transfused mutation-specific CD4+ T cells after chemotherapy failure, and the tumor achieved partial remission 7 months later (78). In addition, some clinical studies on ACT are in progress, such as clinical trial of security and effectiveness of the CAR-T to treat intrahepatic cholangiocarcinoma (NCT03633773). The fourth generation CAR-T cells have been confirmed in the tissue model *in vitro* by targeting CD133 (83). Since more than 50% of cholangiocarcinoma express CD133, the fourth-generation CAR-T cell therapy targeting this target is of great significance for patients with CCA (83).

There are also some early cell therapies, such as lymphokine activated killer cell (LAK) therapy and cytokine induced killer cell (CIK) therapy. LAK treatment works by stimulating the immunocompetent cells of peripheral blood lymphocytes with interleukin-2 (IL-2). That is a mixture of lymphocytes, including T lymphocytes and NK cells. *In vitro*, LAK cells have antigen-independent killing effect on tumor cells. They could kill tumor cells not only by recognizing the surface structure of target cells, but also by secreting cytokines. The mechanism of this target killing is very similar to that of NK cells. This therapeutic effect has been well demonstrated in a variety of malignancies (84). Compared with LAK cells, CIK cells have many advantages: faster proliferation rate, wider tumor killing spectrum, higher tumor killing activity, sensitive to multi drug-resistant cancer cells, less toxic to normal bone marrow hematopoietic precursor cells, can resist the apoptosis of effector cells caused by cancer cells (84). Due to CIK cells originate from the venous blood of sick patients or fitness people, it is relatively easy to culture and expand. At present, CIK cells are widely used in adjuvant treatment of tumors (85). According to this, a great number of clinical experiments were carried out to treat lots of tumors, such as kidney cancer Hodgkin’s lymphoma, leukemia and liver cancer. However, there are few studies on the clinical application of CIK cells in CCA. In one study, by using CCA transplantation of SCID mouse models researchers have found that human CIK cells, composed of CD3+T cells and CD3+/CD56+T cells, can reduce the cell growth (86). In addition, for CCA cells, CIK cells which expressing inducible co-stimulator had the cytotoxic effect (87).

### 4.2 Treatment with vaccines

In addition to cell transplantation, there are also tumor vaccines in the cell therapy of cholangiocarcinoma. Tumor vaccine is extracted from allogeneic or autologous cancer cells, and its components include tumor associated antigen (TAA) or tumor specific antigen (TSA). Its role is to stimulate acquired immune response to attack tumor cells, eliminate the immunosuppression condition result from tumor products, enhance the vaccine potential of TAA, and increase the autoimmunity to attack tumors. It can generally be grouped in four broad categories (tumor cell vaccine, gene vaccine, polypeptide vaccine and dendritic cell (DC) vaccine) by the production source of cancer vaccine.

In a retrospective study, 65 advanced CCA patients received DC vaccine treatment at the same time, 15% of them were relatively stable after 6 months of treatment and had good tolerance to the vaccine (88). A recent study also found that DC vaccine combined with chemotherapy can improve the prognosis of CCA patients more than DC vaccine alone (89). One study has found some distinct antigens, such as FCGR1A, TRRAP and CD247, which can be used to manufacture anti-cholangiocarcinoma mRNA vaccine (90). Other studies have found that two antigens that CCA is more sensitive to are mucin protein 1 (MUC1) and wilm’s tumor protein 1 (WT1) (91, 92). A phase I study result of MUC1 vaccine in the patients with biliary cancer also showed that the vaccine was safe (93). A clinical research using WT1 peptide vaccine combined with gemcitabine in the treatment of biliary cancer included 8 patients with advanced CCA, and found that the tumor control rate of biliary cancer was close to 50% (94). In some case reports and prior clinical experiments, vaccines originated from peptide and DC vaccines have shown positive clinical results (95). In a phase 1 study, nine patients with unresectable BTC were treated with vaccines from four polypeptides, including LY6K, DEPDC1, IMP3, and TTK protein kinase, and the 4 peptides were shown to be extremely effective. What’s more, DEPDC1 and LY6K are very promising candidates for causing an obvious cytotoxic lymphocyte (CTL) reaction, and thereby improving the PFS and OS (96). In one research, thirty-six iCCA patients were injected with autologous tumor lysate added DC vaccine, and activated T cells were transferred at the same time. The OS and median PFS of patients receiving adjuvant treatment with tumor vaccine were 31.9 months and 18.3 months respectively, which were significantly improved compared with 17.4 months and 7.7 months of patients receiving surgery alone (97). DC vaccine would be an acceptable and excellent treatment to prevent postoperative recurrence and improve survival rate of CCA patients.

However, according to the current research conclusion, for most tumors, including cholangiocarcinoma, the single therapy with cancer vaccine usually cannot achieve good clinical effect. Because cancer has multiple immune escape mechanisms, the efficacy of single cancer vaccine therapy is relatively low, and any cancer vaccine alone cannot achieve the expected efficacy. In addition, it is often difficult to evaluate the clinical efficacy of cancer vaccines in patients with cholangiocarcinoma, because the immune status of these patients is often seriously affected by their exposure to other treatments such as surgery, radiotherapy and chemotherapy. The difficulty in evaluating the efficacy further hinders the development of new cancer vaccine drugs (76). The impact of tumor vaccine on CCA patients needs further research and verification. But it is believed that tumor vaccine can be considered as adjuvant therapy for advanced CCA.

## 5 Summary

Immune checkpoint monoclonal antibody plays the role anti-carcinoma by strengthening the now available immune system, but to some extent, it cannot promote immunocytes to target tumors. Cancer vaccine eliminates tumor cells by stimulating unique immune response, but the curative effect is not excellent. However, ACT can directly kill tumors or stimulate the immune response of the body to kill tumor cells, but this research direction is still in the initial stage. The specificity and targeting of the therapy are the research focus at present and the advanced direction in the future. As a matter of fact, we deeply think that researches are needed to assess the clinical application of cell therapy for the treatment of CCA. There is still a long way to go to treat cholangiocarcinoma with this method.

**Figure 1:**
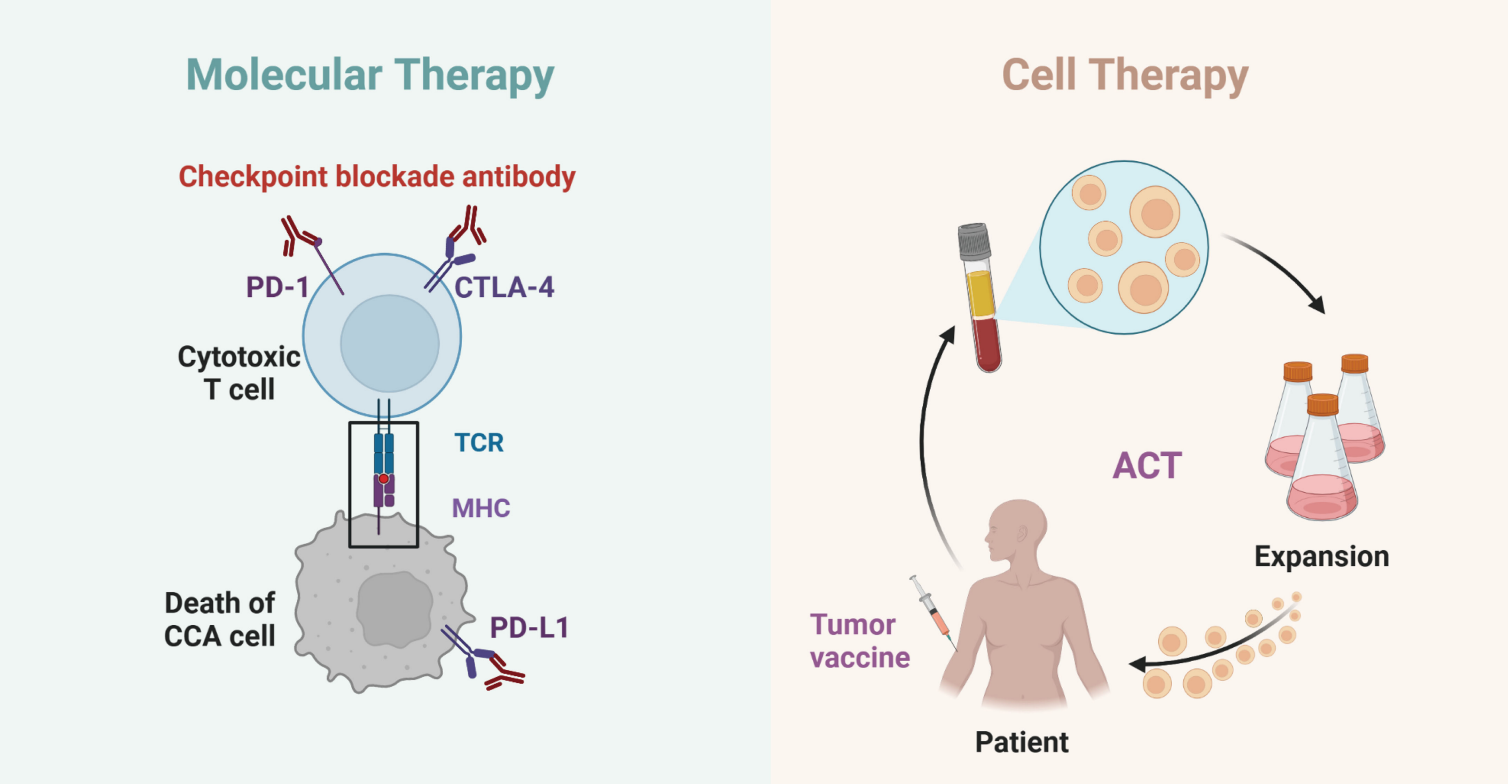
Schematics of molecular and cell therapy in cholangiocarcinoma. The interaction of PD-1/PD-L1 and CTLA4 with cholangiocarcinoma immunotherapy (left); The interaction of tumor vaccine and ACT with cholangiocarcinoma immunotherapy (right).

The treatment of cholangiocarcinoma is complex and difficult, immunotherapy provides us with a new vision in this sphere. In this article, we summarized four immunotherapeutic strategies related to cholangiocarcinoma (**Table 2**, **Figure 1**). We hope that the existing and ongoing studies can be used as a reference for the immunotherapy of cholangiocarcinoma to further enhance the efficacy of immunotherapy and mitigate adverse effects during treatment. However, according to the conclusions of various studies we have obtained, the immunotherapy methods for CCA have their own advantages and disadvantages, and not one scheme can solve a large class of problems. In addition, we still need to note that compared with other tumors, CCA is highly heterogeneous, and the application of immunotherapy in patients with CCA varies significantly from individual to individual. Moreover, there is currently a lack of very specific targets for immunotherapy of CCA, which leads to the slow progress compared with other diseases. Of course, researchers have also made many attempts in these areas, especially immunotherapy combined with targeted therapy and chemotherapy, or local treatment of CCA, which may open up new options and further expand the treatment scope of CCA. We believe that, in the future, with a deeper understanding of the immunotherapy model for cholangiocarcinoma, these immunotherapy methods and immune combination therapies will provide new ideas for the treatment and management of cholangiocarcinoma.

**Table 2:** Some immunotherapeutic strategies currently used in clinical trials to treat CCA.

Immunotherapeutic strategy	Mode of Application	Mechanism
Molecular Therapy	PD-1/PD-L1 inhibitor CTLA4 inhibitor	Relieve the immunosuppression caused by tumor and improve the killing effect on tumor
Cell Therapy	TIL, CAR-T, TCR-T	It can directly kill tumor cells or stimulate the immune response of the body by feeding tumor patients with anti-tumor immune cells cultured or expanded *in vitro*
	LAK, CIK	Stimulate T lymphocytes or antigen presenting cells to enhance antigen presenting process
	MUC1, WT1	With tumor specific antigen or tumor related antigen, it can stimulate specific immune function to attack tumor cells

## Author contributions

L-MZ and A-DS sort out the references. L-MZ, A-DS and Y-CT wrote the manuscript. YY, Z-LL, X-QH and LZ-S read the article and offered some modification comments. Y-CT and Z-LZ devoted to supervising the work and improved the original manuscript. All authors contributed to the article and approved the submitted version.

## Glossary

**Table:**

CCA	cholangiocarcinoma
iCCA	intrahepatic cholangiocarcinoma
pCCA	perihilar cholangiocarcinoma
dCCA	distal cholangiocarcinoma
ACT	adoptive cell therapy
CAR-T	chimeric antigen receptor T-cell
CR	complete remission
DC	dendritic cell
PFS	progression free survival
CIK	cytokine induced killer cell
TILs	tumor-infiltrating lymphocytes
LAK	lymphokine activated killer cell
MMR	mismatch repair
MSI	microsatellite instability
ORR	objective response rates
PD-1	programmed cell death protein-1
CTLA-4	cytotoxic T-lymphocyte antigen-4
PD-L1	programmed cell death protein ligand 1
TKI	tyrosine kinase inhibitor
PR	partial remission
SBRT	stereotactic body radiation therapy
TAA	tumor associated antigen
TMB	tumor mutational burden
TAMs	tumor-associated macrophages
TSA	tumor specific antigen
Treg	regulatory T cell
APC	antigen presenting cell
TME	tumor microenvironment
